# Gender differences in perceived stress and coping among college students

**DOI:** 10.1371/journal.pone.0255634

**Published:** 2021-08-12

**Authors:** B. Sue Graves, Michael E. Hall, Carolyn Dias-Karch, Michael H. Haischer, Christine Apter

**Affiliations:** 1 Department of Exercise Science and Health Promotion Department, College of Science, Florida Atlantic University, Boca Raton, Florida, United States of America; 2 Athletic and Human Performance Research Center, Marquette University, Milwaukee, Wisconsin, United States of America; 3 Program in Exercise Science–Department of Physical Therapy, College of Health Sciences, Marquette University, Milwaukee, Wisconsin, United States of America; 4 Campus Recreation Department, Florida Atlantic University, Boca Raton, Florida, United States of America; University of Westminster, UNITED KINGDOM

## Abstract

**Background:**

Many college students register each semester for courses, leading to productive careers and fulfilled lives. During this time, the students have to manage many stressors stemming from academic, personal, and, sometimes, work lives. Students, who lack appropriate stress management skills, may find it difficult to balance these responsibilities.

**Objectives:**

This study examined stress, coping mechanisms, and gender differences in undergraduate students towards the end of the semester.

**Design and method:**

University students (n = 448) enrolled in three different undergraduate exercise science courses were assessed. Two instruments, the Perceived Stress Scale and Brief Cope, were administered during the twelfth week of the semester, four weeks prior to final exams. T-tests were used to detect gender differences for the stress levels and coping strategies.

**Results:**

Overall, females indicated higher levels of stress than their male counterparts. Gender differences were evident in both coping dimensions and individual coping strategies used. Females were found to utilize the emotion-focused coping dimension and endorsed the use of four coping strategies more often than males. These included self-distraction, emotional support, instrumental support, and venting.

**Conclusions:**

This research adds to the existing literature by illuminating the level of perceived stress and different coping strategies used by undergraduate female and male students. In turn, students may need educational interventions to develop effective and healthy coping strategies to last a lifetime. Faculty and other university officials may want to highlight and understand these various factors to protect the students’ wellbeing in their classes.

## Introduction

College students are challenged with having to keep up with the high demands required to thrive in the university environment. To meet these demands, students must be able to work and function under pressure. Generally, stressors are derived from academia load, classroom environment, faculty interaction, illness, and emotional concerns outside of the classroom [1]. Often, these high-pressured lifestyles can lead to higher stress levels. Additionally, college students undergo a developmental transition from adolescence to adulthood, which can be a challenge and leaves them more susceptible to mental health issues [2].

University students may be unprepared to face additional stressors associated with family, social, academic and financial burdens unique to this population. Growing interest in the use of mind-body approaches for dealing with stress in a healthy way has prompted mounting research in these techniques, and the increase in popularity of activity and general health classes offers an opportunity for valuable research. Stress further impacts students by negatively affecting a student’s concept of self, e.g., self-worth, general health and immune factors [3].

According to the Spring 2019 Health Assessment by the American College of Health Association [4], 34.2% of undergraduate college students had indicated the top impediment to learning was stress, with 45.3% having more than average stress, which may indicate an individual could be more prone to certain illnesses and accidents. This stressful environment has left college students vulnerable to mental health problems, such as anxiety, depression, self-harm, and suicidality [4]. Studies have measured academic performance [5], but not coping skills [6, 7]. Three additional studies utilized both the Perceived Stress Scale (PSS) and Brief COPE for studying stress among college students [8–10]. According to another study [11], university courses would have a significant effect on mitigating stress levels. Other researchers [12] examined the effects that specific exercise courses had on stress levels and found increased mindfulness that resulted from these courses could account for perceived stress changes.

Several other studies found varying differences in female and male college students. Ng and Jeffrey [13] discovered females were more likely to feel as though they experienced higher levels of stress, which was in agreement with Thawabin and Quaisy [14]. In addition, female students reported stress-related issues, such as low self-esteem, pressure from exams, and depression [14]. Higher levels of general and academic stress were also shown to be greater in female students than their male counterparts [15, 16]. However, no gender differences in coping with stress were found [17], whereas other researchers did find relationships between gender and the particular coping mechanism chosen, but agreed the results were not consistent [18]. Another study [8] concluded more similarities existed than differences in perceived stress with men and women, but more investigation regarding those differences were warranted. Two other studies [6, 19] did not have any total perceived stress differences in their college populations, but these authors did not separate their findings by gender. As illustrated, the current literature offers inconsistent findings regarding gender relative to perceived levels of stress.

Concerning coping, researchers have designated coping strategies into various categories, i.e., adaptive vs. maladaptive; active vs. passive; positive vs. negative; problem-focused vs. emotion focused [20]. Active coping refers to purposeful ways to deal with problems and seek comfort and social support, while passive coping is straying away from social support, such as, self-imposed social isolation [21]. The use of active-coping strategies has been associated with higher self-esteem, while the use of passive-coping strategies has been associated with lower self-esteem and lower perceived general well-being [22]. Young adults, when compared to older adults, did have a tendency to utilize more passive coping mechanisms [20, 23]. Other researchers [24] found higher levels of anxiety in a younger adult population when frequently using passive coping mechanisms, such as substance use, avoidance, and self-blaming. Gender, age, and class standing at a university may also relate to passive coping issues [25].

Active emotional regulation abilities can be similar to mindfulness, describing an individual who can recognize and acknowledge personal thoughts and feelings. However, this type of individual has been shown to primarily use intellectual and behavioral action responses, when dealing with stressful emotional experiences to progress towards personal goals [26]. Important implications to support college students in adjustment to transition into classes, especially at the beginning of college, focusing on being resilient and how active emotions, may be a positive or negative influence [27].

Gender differences in coping strategies have been inconclusive because of varying results. For example, these gender differences did not exist in some previous studies [28, 29]. However, females have reported using more problem-focused coping strategies [30] in regard to social support [31, 32], but another study noted that men used these coping strategies more than females [30]. In contrast, additional studies have found that males used more emotion-focused strategies than females [33, 34]. However, this too has been contradicted by other studies that concluded females used more emotion-focused coping strategies than males [35, 36]. Hence, collectively these aforementioned studies indicate that a widely accepted consensus on gender-specific coping strategies differ.

The objective of this present study was to measure both the psychological perception of stress and to evaluate gender differences in coping strategy endorsement. We hypothesized that gender would influence stress levels and utilization of specific coping strategies.

## Materials and methods

### Ethics statement

The research study protocol was approved by the Florida Atlantic University Institutional Review Board (IRB) prior to beginning the study. A written informed consent was obtained from each participant before enrolling.

### Participants

Recruitment of participants was through various sections of the three undergraduate college courses. Yoga, Pilates, and Health Fitness for Life (HFL). The instructors were sent a recruitment letter to read to their classes. A convenience sampling design was utilized to collect the self-reported data from the students. This student sample consisted of university students (n = 448) from a large, suburban, public university in Boca Raton, Florida. The data collection was during the twelfth week of classes, four weeks prior to final exams. Students were currently enrolled in one of the following college courses in the Department of Exercise Science and Health Promotion, Yoga, Pilates or Health Fitness for Life.

### Procedure

Once the instructor gave permission for the recruitment to occur within the class, members of the research team presented the nature of the research to the students. Willing participants signed an adult consent and University Health History Forms before completing the two surveys at the end of their specific class that same day. The students had 30 minutes to complete the Perceived Stress Scale (PSS) and Brief COPE for measurements.

### Measures

Basic demographics were age, gender and class level (Table 1). The Perceived Stress Scale (PSS) measured the degree to which one’s life situations are appraised as stressful [37]. The subjective nature of this measurement instrument has been used as a predictor of health-related outcomes [37]. The survey allowed respondents to report the unpredictability and uncontrollability of their own lives, taking into consideration personality and compounding events [37]. The survey consisted of a brief ten-item questionnaire that rated each item on a scale from 0–4, zero (0) indicating where a person never thought or felt a certain way to four (4) indicating very often [37]. In an initial study, PSS reliability statistics for three groups: freshmen, residing in dormitories; enrolled in an introductory psychology course; and enrolled in a smoking cessation program reported Cronbach alpha coefficients of .84, .85, and .86 respectively [37]. For our study, the stress levels were distributed into three categories of their total scores (mild, 0 to13; moderate, 14–26; and severe 27–40).

**Table 1:** Socio-demographic characteristics of the study sample.

Variables	
N = 448	
AGE	N = 438*
Mean AGE(SD)	20.16 (2.102)
Gender	N = 445**
Male	159 (35.5)
Female	286 (63.8)
Level***	N = 394
Freshman	128 (28.6)
Sophomore	126 (28.1)
Junior	69 (15.4)
Senior	71 (15.8)

*10 missing

**3 missing (.7%)

***missing 54 (12.1%).

In addition, the Brief COPE questionnaire, comprised of twenty-eight questions, was administered to measure coping skills utilized to manage stress, i.e., self-distraction, active coping, emotional support, instrumental support, venting, positive reframing, planning, humor, acceptance and religion [38]. The fourteen subscales of the Brief COPE were categorized into one of three coping dimensions: Problem-focused coping; Emotion-focused coping; and Avoidant coping. Each question was rated on a scale of 1–4, 1 indicated “doesn’t do at all”, to 4 “does a lot” [38]. Twelve items were summed to determine coping skill levels. These scores ranged from 0 to 36. The higher the score for the student indicated more perceived stress and anxiety. The Brief COPE index measured coping skills, as a secondary outcome. Using a sample of Hurricane Andrew survivors in South Florida, reliability coefficients exceeded .50 for the Brief COPE scale [38]. Both PSS and Brief COPE were reported as valid and reliable surveys [32, 37].

Sociodemographic data and distribution of coping and stress level variables within the sample describing the student population were initially analyzed to produce descriptive statistics. Independent t-tests evaluated gender differences in Total PSS score, and across the three cut-off points representing “mild”, “moderate”, and “severe” stress. T-tests were again conducted to determine if gender differences existed for coping style endorsement, as well as the major coping dimensions. The data analyses were completed using SPSS statistical software (version 27, SPSS Inc., Chicago, IL).

## Results

Various independent t-tests compared the differences in Total PSS scores, as well as the established stress level categories between males and females. Overall, gender stress levels differences did exist towards the end of the semester. The females reported higher total PSS than their male counterparts. In addition, more female students reported “moderate” levels of stress than male students (Table 2). There was a significant (p<0.05) gender difference on endorsement for four coping strategies, i.e., self-distractions, emotional support, instrumental support and venting, more so than the males (Table 3). Among the major dimensions of coping, the female students utilized more emotion-focused coping strategies than their male counterparts did. No gender differences in problem-focused or avoidant coping strategies were found.

**Table 2:** Distribution of stress levels in college students.

	Total Sample	Males	Females	t	p
	N (%)	N (%)	N (%)		
Stress Category					
Mild Stress	6 (1.4)	3 (1.9)	3 (1.1)	.609	.106
Moderate Stress	348 (82.3)	140 (90.9)	208 (77.3)	-4.805	.00*
Severe Stress	69 (13.3)	11 (7.1)	58 (21.6)	-.967	.337

* p < .001.

**Table 3:** Differences between male and female students on coping styles.

	Males		Females				
	M	SD	M	SD	t	p	d
Coping Styles							
**Self-Distraction**	5.28	1.72	5.69	1.67	-2.359	**.019**	-.240
Active Coping	6.03	1.45	5.81	1.57	1.378	.169	.139
Denial	2.78	1.33	2.84	1.37	-0.401	.689	-.041
Substance Use	2.94	1.61	2.69	1.36	1.633*	.104	.173
**Emotional Support**	4.33	1.86	5.08	1.78	-4.082	**.000**	-.415
**Instrumental Support**	4.5	1.78	5.14	1.91	-3.315	**.001**	-.336
Behavioral Disengagement	2.75	1.24	2.97	1.31	-1.742	.082	-.177
**Venting**	3.56	1.51	4.09	1.55	-3.409	**.001**	-.346
Positive Reframing	5.58	1.63	5.88	1.67	-1.823	.069	-.185
Planning	5.58	1.66	5.67	1.67	-0.580	.562	-.059
Humor	4.43	1.86	4.24	1.92	1.036	.301	.105
Acceptance	5.51	1.71	5.52	1.67	-0.079	.937	-.008
Religion	4.33	2.09	4.69	2.20	-1.629	.104	-.165
Self-Blame	4.01	1.69	4.17	1.69	0-.948	.344	-.096
Major Types of Coping							
Problem Focused	16.13	3.85	16.64	4.01	-1.281	.201	-.130
**Emotion Focused**	31.64	7.45	33.60	7.23	-2.609	**.009**	-.268
Avoidant Coping	13.74	4.11	14.18	3.80	-1.113	.266	-.114

* Sig. Levene’s.

## Discussion

This study’s purpose was to investigate the presence of gender differences on stress levels and coping strategies towards the end of the semester of undergraduate students. These students were officially registered for either one of two activity exercise classes (Yoga or Pilates) or one lecture course (Health Fitness for Life).

The present study found significant gender differences in perceived stress levels, with the females reporting significantly higher total PSS levels, in addition, more females indicated more moderate levels of stress compared to their male counterparts. Being a female has been positively associated with perceived stress [10], similar to our findings. Brougham, Zail, Mendoza and Miller [39] agreed, too, that, generally, stress levels for college women were higher. This susceptibility to stress levels in females was in agreement with other studies [10, 15, 16, 40–42]. Schmaus, et al. [42] reviewed gender differences in a controlled setting, which found women were possibly more vulnerable to repeated stress exposures when compared to men. Again, Thawabein and Quaisy [14] agreed with Harutyunyan, Musheghyan, Hayrumyan [43] that women had experienced more perceived stress, than male students, similar to our study. Notably, the transition to a college explained the high stress of their first year as students. However, Leong, Bonz, and Zachar’s [44] did not have similar findings as ours, in regards to stress, since they observed no significance in either in their sample of highly functioning college freshmen at an Ivy League institution. The researchers concluded these students parents tended to be more highly educated than the parents at larger state universities, a possible explanation for their findings.

Coping has been defined as mental processes and behaviors which individuals use in order to manage stressful situations, internally and externally [45, 46]. The Brief COPE consists of fourteen dimensions with two items each (28 items in total), including adaptive coping strategies, e.g., active coping, planning, positive reframing, acceptance, humour, religion, seeking emotional and instrumental support, and maladaptive coping techniques, e.g., self-distraction, denial, venting, substance use, behavioural disengagement, and self-blame [38, 47]. Problem focused categories were active, planning and instrumental support. Emotion focused were acceptance, humor, emotional support, venting, religion, positive reframing, self-blame. When dealing with stress and accompanying stressors, using these coping mechanisms are essential.

The females in our study endorsed four specific coping styles (self-distraction, emotional support, instrumental support and venting) compared to males. One study [48], also indicated several notable differences that were found in coping strategies utilized while dealing with stress between genders. While female university students have reported more stress [4, 39], similar to our study, a review of the coping literature, regarding gender differences, tended to be less specific. Thus, gender was the important component in this study, which should be considered, when being mindful of university student stress reduction.

Self-distraction may be perceived as an effective coping mechanism among the students. Possibly, the individual does perceive relief in the form of temporal escape from the stressor, however, this relief does little to resolve the actual stressor. Another study [49] strongly suggested the college student, instead of concentrating on their stress-related health issues, should use interventions that would develop more mindfulness to reduce anxiety levels, focus and memory. Females had significant differences when compared to their male counterparts with self-distraction [50]. Individuals using self-distraction coping strategies may provide immediate relief, which reinforces the use of that strategy. The individual may adopt these types of coping methods to manage their levels of stress because of a lack of another positive coping method. This stress may result from having issues with perceived control of the situation. Further examination of self-distraction, which can be positive reframing mechanisms (active coping), in relationship with perceived stress that allows for a better understanding of the various ways college students dealt with stressors [8, 51]. While we cannot conclude a causal relationship, we can identify areas to address with focused stress management interventions.

Another study [51] found final year female medical students utilized emotional-coping strategies, specifically, sought emotional support, to manage their stress, similar to our study. These strategies can build on the existing adaptive emotion-focused approaches used by these students. When individuals, such as these college females, find themselves in undesirable situations (stressful), they may seek to assign blame to internal or external sources. However, the college males seek much lower levels of support, since they either may lack the social network or may not have developed those skills [8]. Additionally, one study [52] did agree with the gender differences in our current study that females used more emotion-focused type of coping than their male counterparts did. Both genders may develop the ability to effectively connect and regulate their emotions that contribute to a reduction in stress and continuing relationships [53].

Instrumental support was another higher emotional support coping mechanism the females in our study used. Instrumental support, in regards to perceived stress, has been considered a positive support mechanism and has related to subjective well-being, e.g., by listening or providing tangible assistance to another individual. A previously mentioned study [8] also found women used instrumental support as a significant coping predictor than men consistent with other studies [31, 54]. In addition, another study [55] agreed with our study that females use instrumental support more frequently than males.

Another coping strategy the females used in our study was venting. This style allows an individual to express anger, and may result in a cathartic relief from the immediate effects of stressful situations. As a coping strategy, venting is considered passive, or ineffective at managing stress and may, in fact, intensify one’s stress level [56]. Visker, Rider and Humphers-Ginther [57] acknowledged that it was generally acceptable to discuss personal feelings about stress, and excessive voicing of displeasure (venting) should be discouraged. However, venting one’s frustrations is often associated with a cathartic relief of stress symptoms [58]. Xia, Ding, Hollon and Yi [59] suggested venting reliance as a coping strategy increased the amount of interpersonal stress through recalling stressful events during the outburst. While the individual may experience a subsiding of the symptoms, this relief is often fleeting and, actually, results in an exacerbation of stress [60]. Venting, by crying, may leave a male more uncomfortable and distressed than if a female did the same [54]. Another suggestion by these authors is that males may adopt more anger in their expressions than women, who may express through tears. Also, similar to our findings, another study [44] did find females scored significantly higher on venting of emotions. Agreeing with our study, other researchers [61] did show a gender difference with venting, but the subjects were a much older population and had kidney disease. However, venting was not always a coping strategy used by females, as previously noted [8], that found males used venting, contrary to our finding.

Among the major dimensions of coping, the females used emotion-focused approaches greater than males. Emotional-focused coping typically brings about short-term relief of stress but, often, does not result in situational change, i.e., resolution of the stressor. Generally, college females (more than college males) have a tendency to rely more on this type of support, since women may feel they need this support [51, 62]. Both genders may develop the ability to effectively connect and regulate their emotions that contribute to a reduction in stress and continuing relationships [53]. However, males have tended to use problem-focused coping in stressful times, according to another study [44], but did not have the same findings as this study.

## Conclusion

All the data collected were based on participant self-report, which can be vulnerable to sources of bias, such as societal attraction, the individual’s memory, and attentiveness. Another reflection was that the majority of the sample were female. However, university students, with the majority being female were included in similar studies [12, 25, 27, 63]. For future research, randomized sampling procedures, extended time for the study, and possibly implementing additional coping strategies into the specific courses, at the beginning of the university semester, should possibly be included.

This study’s findings have provided pertinent information in order to reduce stress, more specific to gender. Possibly, more effective stress management and adaptive sessions could have more emphasis incorporated into classes, especially at the freshman and/or sophomore level. This evidence can also be used to apply to the design of future studies and possible guidance in undergraduate students, again, specific to gender.

Therefore, this current study did provide support for gender differences in a university students’ ability to actually cope with total perceived stress. Serious consideration needs to be given as to how male and female students will be able to separately develop successful life-long coping skills and to handle stressful situations, since both are using different coping mechanisms to deal with their stress. The information, within this study, did provide the way that perceived stress and different coping mechanisms vary with gender differences and the coping strategies students utilize.

The majority of undergraduate female students experienced medium to higher levels of stress. The males experienced much lower perceived stress levels than the females. Additional research will be needed to continue to discover specific gender differences in stress levels and coping mechanisms concerning undergraduate students. The results in our study may be useful for university faculty and, possibly, administration in order to create stress-reduction strategies. Since gender disparities seem to exist in perceived and actual stress levels, further investigation is warranted to handle reduction of student stress and development of coping strategies.

## Supporting information

S1 Dataset This is the study dataset file. (CSV)

## References

[1] Kizhakkeveettil A, Vosko A, Brash M, Philips M. Perceived stress and fatigue among students in a doctor of chiropractic training program. J Chiropr Educ. 2017;31(1):8–13. doi: 10.7899/JCE-15-27 27552030 PMC5345784

[2] Zhang M, Zhang J, Zhang F, Zhang L, Feng D. Prevalence of psychological distress and the effects of resilience and perceived social support among Chinese college students: Does gender make a difference? Psychiatry Res. 2018;267:409–413. doi: 10.1016/j.psychres.2018.06.038 29960938

[3] Largo-Wight E, Peterson P, Chen W. Perceived problem solving, stress, and health among college students. Am J Health Beh. 2005;29(4):360–370. 10.5993/ajhb.29.4.8. 16006233

[4] American College of Health Association-National College Health Assessment [Internet]. Silver Spring, MD; c2019 [cited 2021 Apr 6]. Reference Group Executive Summary. Available (2019). from: https://www.acha.org/documents/ncha/NCHA-II_SPRING_2019_US_REFERENCE_GROUP_EXECUTIVE_SUMMARY.pdf.

[5] Kauts A, Sharma N. Effect of yoga on academic performance in relation to stress. Int J Yoga 2009;2(1):39–43. doi: 10.4103/0973-6131.53860 21234215 PMC3017967

[6] Deckro GR, Ballinger KM, Hoyt M, Wilcher M, Dusek J, Myers P, et al. The evaluation of a mind/body intervention to reduce psychological distress and perceived stress in college students. J Am Coll Health. 2002;50(6):281–287. doi: 10.1080/07448480209603446 12701653

[7] Vidic Z, Cherup N. Mindfulness in classroom: Effect of a mindfulness-based relaxation class on college students’ stress, resilience, self-efficacy and perfectionism. Coll Stud J. 2019;53(1):130–142.

[8] Eisenbarth CA. Coping with stress: Gender differences among college students. Coll Student J. 2019;53(2):151–162.

[9] Furman M, Joseph N, Miller-Perrin C. Associations between coping strategies, perceived stress, and health indicators. Psi Chi J Psychol Res. 2018;23(1):61–72. 10.24839/2325-7342.jn23.1.61.

[10] Deatherage S, Servaty-Seib H, Aksoz I. Stress, coping, and internet use of college students. J Am Coll Health. 2014;62(1):40–46. doi: 10.1080/07448481.2013.843536 24313695

[11] Donahoe-Fillmore B, Fisher M, Brahler C. The effects of home-based Pilates in healthy college-aged women. J Womens Health Phys Therap. 2015;39(2):83–94. 10.1097/jwh.0000000000000031.

[12] Caldwell K, Harrison M, Adams M, Quin R, Greeson J. Developing mindfulness in college students through movement-based courses: effects on self-regulatory self-efficacy, mood, stress, and sleep quality. J Am Coll Health 2010;58(5):433–442. doi: 10.1080/07448480903540481 20304755 PMC2879280

[13] Ng DM. Jeffrey RW. Relationship between perceived stress and health behaviours in a sample of working adults. Health Psychol. 2003;22(6):638–642. doi: 10.1037/0278-6133.22.6.638 14640862

[14] Thawabien AM, Qaisy LM. Assessing stress among university students. American Int J Contemp Res. 2012;2(2):110–116.

[15] Backović DV, Živojinovic JI, Maksimović J, Maksimović M. Gender differences in academic stress and burnout among medical students in final years of education. Psychiatria Danubina. 2012;24(2):75–181. 22706416

[16] Rahardjo W, Juneman J, Setiani Y. Computer anxiety, academic stress, and academic procrastination on college students, J Educ Learn. 2013;7(3):147–152.

[17] Donaldson D, Prinstein MJ, Danovsky M, Spirito A. A pattern of children’s coping with life stress: Implications for clinicians. Am J Orthopsychiatry 2000;70(3):351–359. doi: 10.1037/h0087689 10953781

[18] Thoits PA. Stress, coping and social support processes: Where are we? What next? J Health and Soc Behav 1995;35:53–79. 7560850

[19] Jones DR, Lehman BJ, Noriega A, Dinnel DL. The effects of a short-term mindfulness meditation intervention on coping flexibility. Anxiety Stress Coping. 2019;32(4):347–361. doi: 10.1080/10615806.2019.1596672 30929458 PMC6900869

[20] Hunt S, Wisocki P, Yanko J. Worry and use of coping strategies among older and younger adults. J Anxiety Disord. 2003;17(5):547–560. doi: 10.1016/s0887-6185(02)00229-3 12941365

[21] Ashkar PJ, Kenny DT. Views from the inside: Young offenders’ subjective experiences of incarceration. Intl J Offender Ther Comp Criminol. 2008;52(5):584–597. doi: 10.1177/0306624X08314181 18296631

[22] Barendregt C, Laan A, Bongers I, Nieuwenhuizen C. Adolescents in secure residential care: the role of active and passive coping on general well-being and self-esteem. Eur Child & Adolesc Psychiatry. 2015;24(7):845–854. doi: 10.1007/s00787-014-0629-5 25325990

[23] Blanchard-Fields F. Everyday problem solving and emotion. Curr Dir Psychol Sci. 2007;16(1):26–31. 10.1111/j.1467-8721.2007.00469.x.

[24] Mahmoud JS, Staten R, Hall LA, Lennie TA. The relationship among young adult college students’ depression, anxiety, stress, demographics, life satisfaction, and coping styles. Issues Ment Health Nurs. 2012;33(3):149–156. doi: 10.3109/01612840.2011.632708 22364426

[25] Mahmoud JS, Staten R, Lenni TA, Hall LA. The relationships of coping, negative thinking, life satisfaction, social support, and selected demographics with anxiety of young adult college students. J Child Adolesc Psychiatr Nurs. 2015;28(2):97–108. doi: 10.1111/jcap.12109 25939686

[26] Gratz KL, Roemer L. Multidimensional assessment of emotion regulation and dysregulation: development, factor structure, and initial validation of the difficulties in emotion regulation scale. J of Psychopathol Beh Assess. 2004;26(1):41–54.

[27] Finkelstein-Fox L, Park CL, Riley KE. Mindfulness and emotion regulation: promoting well-being during the transition to college. Anxiety Stress Coping. 2018;31(6):639–653. doi: 10.1080/10615806.2018.1518635 30189751

[28] Hamilton S, Fagot BI. Chronic stress and coping styles: A comparison of male and female undergraduates. J Pers Soc Psychol. 1988;55:819–823. doi: 10.1037//0022-3514.55.5.819 3210149

[29] Rosario M, Shinn M, Morch H, Huckabee CB. Gender differences in coping and social supports: Testing socialization and role constraint theories. J Community Psychol 1988;16:55–69.

[30] Ptacek JT, Smith RE, Zanas J. Gender, appraisal, and coping: A longitudinal analysis. J of Personal. 1992;60:747–770.

[31] Matud MP. Gender differences in stress and coping styles. Personal Ind Diff. 2004;37(7):1401–1415.

[32] Carver C, Scheier M, Weintraub JK. Assessing coping strategies: A theoretically based approach. J of Per Soc Psychol. 1989;56:267–283. doi: 10.1037//0022-3514.56.2.267 2926629

[33] Kieffer KM, Cronin C, Gawet D. L. Test and study worry and emotionality in the prediction of college students’ reasons for drinking: An exploratory investigation. J Alcohol Drug Edu 2006;50:57–81.

[34] Sigmon ST, Stanton AL, Snyder CR. Gender differences in coping: A further test of socialization and role constraint theories. Sex Roles 1995;33:565–587.

[35] Eaton RJ, Bradley G. The role of gender and negative affectivity in stressor appraisal and coping selection. Int J Stress Manag. 2008;15: 94–115.

[36] Harju BL, Bolen LM. The effects of optimism on coping and perceived quality of life of students. J Soc Beh Personal. 1998;13:185–200.

[37] Cohen S, Kamarck T, Mermelstein R. A. global measure of perceived stress. J Health Soc Beh.1983;24(4):385–396. 10.2307/2136404 6668417

[38] Carver C. You want to measure coping but your protocol’s too long: Consider the brief cope. Intl J of Behav Med. 1997;4(1):92–100. doi: 10.1207/s15327558ijbm0401_6 16250744

[39] Brougham RR, Zail CM, Mendoza CM, Miller JR. Stress. Sex differences, and coping strategies among college students. Curr Psychol. 2009;38:85–97.

[40] Hall NC, Chipperfield JG, Perry RP, Ruthig JC, Goetz T. Primary and secondary control in academic development: gender-specific implications for stress and health in college students. Anxiety Stress Coping. 2006;19(2):182–210.

[41] Shaw MP, Peart DJ, Fairhead OJW. Perceived stress in university students studying in a further education college. Res Post-Compulsory Edu. 2017;22(3):442–452.

[42] Schmaus BJ, Laubmei KK, Boquiren VM, Herze M, Zakowski SG. Gender and stress: Differential psychophysiological reactivity to stress reexposure in the laboratory, Inter J Psychophysiol 2008;69(2):101–106. doi: 10.1016/j.ijpsycho.2008.03.006 18453025

[43] Harutyunyan A, Musheghyan L, Hayrumyan V. Gender differences in perceived stress level among undergraduate students in Armenia. Eur J Public Health Supplement, 2020;30(5). 10.1093/eurpub/ckaa166.1028.

[44] Leong TL, Bonz MH, Zachar P. Coping styles as predictors of college adjustment among freshmen. Couns Psychol Q. 1997;10(2):221–220.

[45] Folkman S, Lazarus RS. An analysis of coping in a middle-aged community sample. J Health and Soc Beh. 1980;21(3):219–239. 10.2307/2136617. 7410799

[46] Lazarus S, Folkman RS. An analysis of coping in a middle-aged community sample. J Health Soc Beh. 1982;21(3):219–239. 7410799

[47] Gambetta-Tessini K, Marino R, Morgan M, Anderson V. Coping strategies and the Salutogenic Model in future oral health professionals. BMC Med Educ. 2016;16(224):1–8. doi: 10.1186/s12909-016-0740-z 27562194 PMC5000445

[48] Jones K, Mendenhall S, Myers CA. The effects of sex and gender role identity on perceived stress and coping among traditional and nontraditional students. J Am Coll Health 2016;64(3):205–213. doi: 10.1080/07448481.2015.1117462 26730819

[49] Rizer CA, Fagan MH, Kilmon C, Rath L. The role of perceived stress and health beliefs on college students’ intentions to practice mindfulness meditation. Am J Health Educ. 2016;47(1):24–31. 10.1080/19325037.2015.1111176.

[50] Adasi GS, Amponsah KD, Mohammed SM, Yeboah R, Mintah PC. Gender differences in stressors and coping strategies among teacher education students at University of Ghana. J of Educ Learn. 2020;9(2):123–133.

[51] Madhyastha S, Latha KS, Kamath A. Stress and coping among final year medical students. J Psychol Med. 2014;15(2):74–80.

[52] Amin R, Ashadullah M, Sulton S. Perceived stress and coping strategies among undergraduate university students: Role of gender. Bahria J Prof Psychol. 2019;18(1):63–76.

[53] Skowron E A, Wester S, Azen R. Differentiation of self mediates college stress and adjustment. J Couns Devel. 2004;82(1):69–8.

[54] Tamres LK, Janicki D, Helgeson VS. Sex differences in coping behavior: A meta-analytic review and examination of relative doping. Per Soc Psychol Rev. 2002;6(1):2–30.

[55] Garcia JL, Heckman JJ, Ziff A. Gender differences in the benefits of an influential early childhood program. Euro Econ Rev. 2018;109:9–22. doi: 10.1016/j.euroecorev.2018.06.009 30410186 PMC6217989

[56] Kaholodula JK, Antonio MCK, Ing CKT, Hermosura A, Hall KE, Knight R, et al. The effects of perceived racism on psychological distress mediated by venting and disengagement coping in Native Hawaiians. BMC Psychol. 2017;5(1):2–10. doi: 10.1186/s40359-017-0171-6 28081710 PMC5228113

[57] Visker JD, Rider T, Humphers-Ginther A. Ministry-related burnout and stress coping mechanisms among assemblies of God-Ordained clergy in Minnesota. J Relig Health. 2017;56(3):951–961. doi: 10.1007/s10943-016-0295-7 27510527

[58] Bushman BJ. Does venting anger feed or extinguish the flame? Catharsis, rumination, distraction, anger, and aggressive responding. Per and Soc Psychol Bull. 2002;28(6):724–731.

[59] Xia LX, Ding C, Hollon SD, Yi Y. The effects of perceived racism on psychological distress mediated by venting and disengagement coping in Native Hawaiians. Cur Psychol. 2015;34(1):14–25. 10.1186/s40359-017-0171-6 PMC5228113 28081710

[60] Joshanloo M. Fragility of happiness moderates the influence of negative predictors of subjective well-being. Anxiety Stress Coping. 2018;31(2):222–227. doi: 10.1080/10615806.2017.1422094 29298493

[61] Gemmell LA, Terhorst L, Jhamb M, Unruh M, Myaskovsky L, Kester L, et al. Gender and racial differences in stress, coping, and health-related quality of life in chronic kidney disease. J Pain Symptom Manage. 2016;52(6):806–812. doi: 10.1016/j.jpainsymman.2016.05.029 27697565 PMC5156935

[62] Weckwerth AC, Flynn DM. Effect of sex on perceived support and burnout in university students. Coll Student J. 2006;40(2):237–249.

[63] Lemay V, Hoolahan J, Buchanan A. Impact of a yoga and meditation intervention on students’ stress and anxiety levels. Am J of Pharm Educ. 2019;83(5):7001. doi: 10.5688/ajpe7001 31333265 PMC6630857

